# A Multi-Compartment Model of Glioma Response to Fractionated Radiation Therapy Parameterized *via* Time-Resolved Microscopy Data

**DOI:** 10.3389/fonc.2022.811415

**Published:** 2022-02-04

**Authors:** Junyan Liu, David A. Hormuth, Jianchen Yang, Thomas E. Yankeelov

**Affiliations:** ^1^ Department of Biomedical Engineering, The University of Texas at Austin, Austin, TX, United States; ^2^ Oden Institute for Computational Engineering and Sciences, The University of Texas at Austin, Austin, TX, United States; ^3^ Livestrong Cancer Institutes, The University of Texas at Austin, Austin, TX, United States; ^4^ Department of Diagnostic Medicine, The University of Texas at Austin, Austin, TX, United States; ^5^ Department of Oncology, The University of Texas at Austin, Austin, TX, United States; ^6^ Department of Imaging Physics, The University of Texas MD Anderson Cancer Center, Houston, TX, United States

**Keywords:** radiobiology, glioma, computational biology, mathematical modeling, oncology, brain cancer cell

## Abstract

**Purpose:**

Conventional radiobiology models, including the linear-quadratic model, do not explicitly account for the temporal effects of radiation, thereby making it difficult to make time-resolved predictions of tumor response to fractionated radiation. To overcome this limitation, we propose and validate an experimental-computational approach that predicts the changes in cell number over time in response to fractionated radiation.

**Methods:**

We irradiated 9L and C6 glioma cells with six different fractionation schemes yielding a total dose of either 16 Gy or 20 Gy, and then observed their response *via* time-resolved microscopy. Phase-contrast images and Cytotox Red images (to label dead cells) were collected every 4 to 6 hours up to 330 hours post-radiation. Using 75% of the total data (i.e., 262 9L curves and 211 C6 curves), we calibrated a two-species model describing proliferative and senescent cells. We then applied the calibrated parameters to a validation dataset (the remaining 25% of the data, i.e., 91 9L curves and 74 C6 curves) to predict radiation response. Model predictions were compared to the microscopy measurements using the Pearson correlation coefficient (PCC) and the concordance correlation coefficient (CCC).

**Results:**

For the 9L cells, we observed PCCs and CCCs between the model predictions and validation data of (mean ± standard error) 0.96 ± 0.007 and 0.88 ± 0.013, respectively, across all fractionation schemes. For the C6 cells, we observed PCCs and CCCs between model predictions and the validation data were 0.89 ± 0.008 and 0.75 ± 0.017, respectively, across all fractionation schemes.

**Conclusion:**

By proposing a time-resolved mathematical model of fractionated radiation response that can be experimentally verified *in vitro*, this study is the first to establish a framework for quantitative characterization and prediction of the dynamic radiobiological response of 9L and C6 gliomas to fractionated radiotherapy.

## 1 Introduction

Radiation therapy is a central component of the standard-of-care for treating malignant gliomas (1), especially when the tumor is located near sensitive brain regions with important functions that are unresectable by surgery. Though various dose escalation and fractionation schemes (i.e., hyper- and hypo- fractionation) have been investigated, none have shown definitive improvement on the long-term survival for glioblastoma patients (2). One reason for this limitation is that the efficacy of radiation therapy varies between patients due to heterogeneous radiosensitivity of the cells within each individual’s tumor (3). If there was a mathematical model that could accurately characterize, and predict, the response of tumor cells to radiation therapy with patient-specific data, then there would be the opportunity to optimize the radiation plan for each individual (4). The currently accepted model for evaluating radiation response given a specific dose is the linear quadratic (LQ) model which was originally developed empirically more than 40 years ago (5). The LQ model quantifies the survival fractions of cell colonies given a specific radiation dose and, though it provides a simple, and practical relationship between those two measureables, it is not without its limitations. In particular, the LQ model does not explicitly characterize the temporal changes in tumor cell number; that is the LQ model is not a function of time. Thus, while it can provide accurate predictions of endpoint predictions (6), it is not capable of predicting the temporal dynamics of radiation response. Additionally, interpretation of the two main parameters in the LQ model (alpha and beta) is fraught with difficult, thereby clouding their biological meaning (7). This is despite the now vast biological knowledge that exists regarding DNA repair (8) and radiation-induced cell death pathways (9). To address these two limitations, we previously proposed and validated a mechanism-based time-resolved model (10) to a single-dose treatment. We now seek to extend this model to account for multiple-fraction treatment regimens.

Though a large single-dose of radiation can effectively kill tumor cells, it is rarely used in clinical settings as high doses also cause irreversible cytotoxicity to surrounding healthy tissues. Thus, the notion of delivering a target total dose in “fractions” over an extended period of time was adopted. There are four key conceptions that are frequently kept in mind when designing multi-fraction treatment plans: DNA damage repair, repopulation, cell cycle redistribution, reoxygenation [sometimes referred to as the “Four R”s (11)]. The DNA damage repair mechanisms help the nearby healthy tissue recover between treatment intervals (12), with the hope that the repair mechanisms are erroneous in the tumor cells leading to their eventual cell death after repeated fractions (13). Tumor cell repopulation (i.e., the ability of tumor cells to proliferate between treatment intervals) can undermine radiation efficacy, and thus may require extra fraction and/or total dose to achieve tumor control (14). Cell cycle redistribution increases the average tumor cell killing by allowing radiation-resistant cells in S phase to redistribute into the more sensitive M phase (15). Reoxygenation also enhances radiation damage as radiation can produce free radicals which damage DNA, and this damage can be made permanent by the presence of molecular oxygen [i.e., the ‘oxygen-fixation hypothesis’ (16)]. Additionally, hypoxic regions of a tumor regions may become reoxygenated between fractions (17). Previous modeling work has focused on quantifying the effects of these four “R”s on the endpoint survival fraction by (for example) incorporating an “oxygen enhancement ratio” (18) or repopulation (19) into the LQ model. More recently, several studies have constructed radiation response models that account for temporal changes in hypoxia (20), DNA repair (21), and fractionation (22). Hormuth et al. contributed a tissue-scale model that employed the oxygen enhancement ratio to adjust the radiation efficacy during fractionation treatment and tested model predictions against *in vivo* MRI data (23, 24). Brüningk et al. (25) proposed a cell-scale decision tree model that accounted for conversion between cell cycle compartments after radiation. All these models indicate an increasing interest in mathematically describing the temporal dynamics of radiation response that are not captured by the conventional LQ-based models.

We first propose to extend our previous single-dose model (10) to characterize the radiation response of gliomas to fractionated treatment. The fractionation model explicitly incorporates temporal changes due to DNA damage repair, cell repopulation, and cell cycle effect related to senescence. We then perform *in vitro* microscopy experiments with 9L and C6 cell lines to obtain the radiation response curves collected at high temporal resolution under different treatment schedules and total radiation doses. Our model is then trained on 75% of the total data to calibrate the parameters. Finally, the remaining 25% of data serve as a validation group to assess the model’s predictive accuracy. Our mechanism-based, time-resolved, mathematical model achieves high predictive accuracy across a range of fractionation schedules verified by six different fractionation schemes and both cell lines.

## 2 Materials and Methods

### 2.1 Experiments

#### 2.1.1 Cell Culture

The 9L (American Type Culture Collection, ATCC) and C6 (Sigma Aldrich) cells are cultured according to the manufacturer’s guidelines as previously described (10). The 9L cell line is cultured with Eagle’s minimum essential medium (ATCC, VA), and the C6 cell line is cultured with Ham’s F12 (Corning, NY). Both cell lines media are supplemented with 10% FBS and 2 mM L-Glutamine. 0.2% Plasmocin Prophylactic (*In vivo*gen, CA) is supplied in the media to prevent mycoplasma contamination. The mean passage number of the cells used in the experiments was 50 ± 20. Both 9L and C6 are rat cell lines, but are commonly used for general glioma studies (26, 27).

#### 2.1.2 Radiation Treatment and Imaging

**Figure 1:**
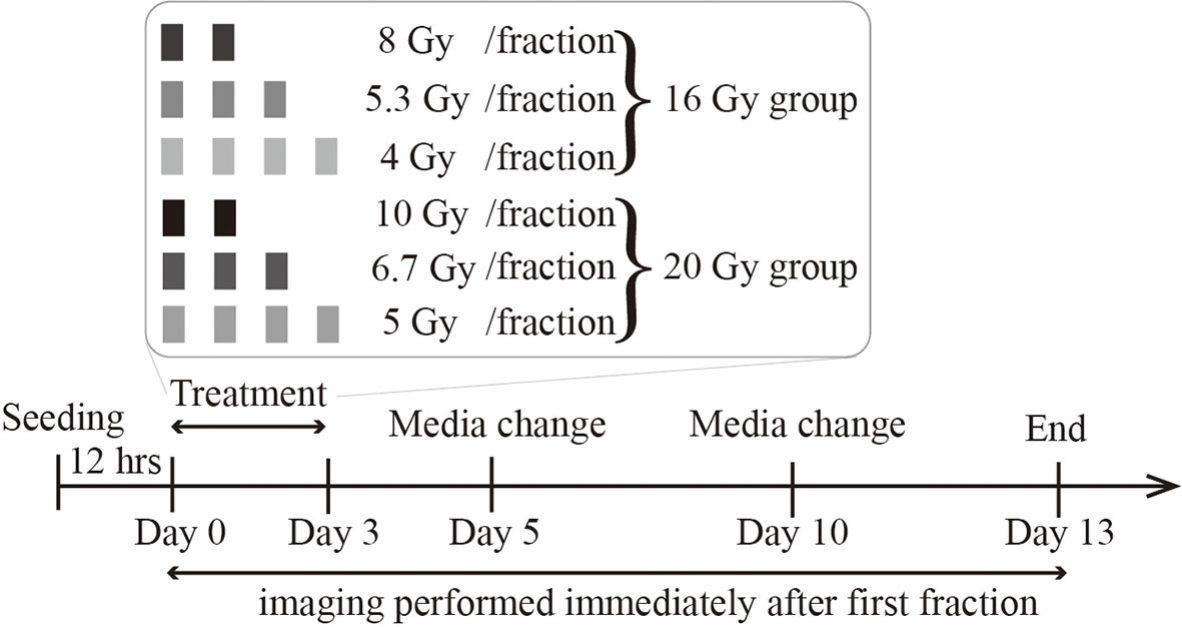
Radiation treatment schedule. Cells are seeded, incubated overnight, and then treated with either a total dose of 16 Gy or 20 Gy. In the 16 Gy total dose group, cells are irradiated with 2 fractions of 8 Gy, 3 fractions of 5.3 Gy, or 4 fractions of 4 Gy. In the 20 Gy total dose group, cells are irradiated with 2 fractions of 10 Gy, 3 fractions of 6.7 Gy, or 4 fractions of 5 Gy. All irradiations are 24 hours apart. The culture media is refreshed every 5 days, and imaging lasts up to two weeks after the initial irradiation.

**Figure 1** illustrates the radiation treatment schedule employed in these studies. 9L and C6 cells were seeded on 96-well plates (Corning, NY) at densities ranging from 3,200 to 32,000 cells/cm^2^ (1,000 to 10,000 cells total per well). To avoid cells reaching the carrying capacity at later timepoints (which can result in cell death due to lack of nutrients and physical space), we do not seed at a confluence higher than 10,000 total cells. The cells are then incubated overnight (~12 hours) to allow for attachment and recovery. Before irradiation, the media is changed and augmented with 250 nM Cytotox Red dye (Cat. *No*. 4632, Essen BioScience, MI), a non-perturbing fluorescent dye to label dead cells. For both the 9L and C6 cell lines, we separate the wells into either a 16 Gy or a 20 Gy total dose group. In the 16 Gy group, we irradiate the cells with either four fractions of 4 Gy, three fractions of 5.3 Gy, or two fractions of 8 Gy with 24-hour intervals between every fraction. In the 20 Gy group, we irradiate cells with four fractions of 5 Gy, three fractions of 6.7 Gy, and two fractions of 10 Gy with 24-hour intervals between every fraction. All radiation is delivered by a CellRad irradiator (Faxitron X-Ray Corp, Wheeling, IL, MA) at a dose rate of 1.5 Gy/min (130 KeV, 5 mA, 0.5 mm aluminum filter). After treatment, phase-contrast images and fluorescent Cytotox Red images (for labeling dead cells) are acquired immediately after the first fraction *via* the Incucyte S3 live imaging system (Essen BioScience, Ann Arbor, MI) with a 4× objective, whole-well imaging mode every four to six hours up to approximately 330 hours post-irradiation. Our media culture, along with the Cytotox Red dye, was refreshed every five days throughout the experiment. To prevent cell loss when refreshing the media in the 96-well plates, we pipetted only the top 80 μl of the total 100 μl per well to minimize the disturbance to attached cells. Live and dead cells were segmented using a semi-automated pipeline consisting of a histogram Otsu-based method followed by a morphology-based cell debris removal [described in detail in (10)].

#### 2.1.3 DSB Repair Kinetics

To quantify radiation-induced DNA double strand breaks (DSB) and repair kinetics, we previously measured the expression level of the γH2AX protein [a commonly used DSB biomarker (28)] *via* flow cytometry after irradiating cells with a single dose of 2, 4, 8, or 16 Gy (see the **Supplementary Materials** of (10) for details). We then used linear interpolation to obtain the DSB repair kinetics for all other doses. The same data is used in this study as described below.

#### 2.1.4 DNA Repair

DNA repair is represented by an exponential decay equation:


(1) $$f_{DSB}(t,D)=e^{-k_{repair}(D)\cdot t}$$

where *f_DSB_* (*t*,*D*) is the fraction of DSBs remaining unrepaired (normalized between 0 and 1) at time, *t*, *k_repair_* (*D*) (in units of *hr^-1^*) is the DSB repair rate at dose per fraction *D*. We use the same *k_repair_* measured from our single-dose treatment study as an estimate of the DSB repair rate during fractionation schedules. This assignment is justified as Mariotti et al. (29) have measured γH2AX under both single and multi-fractionated treatment schemes and showed similar repair kinetics in response to a second dose when cells are given proper time for repair and recovery. Our previous flow cytometry experiments (10) indicate that most (> 80%) DSBs are repaired within 24 hours given the dose range we employ in the experiments. Therefore, this estimation is reasonable.

### 2.2 Mathematical Modeling of Cell Growth and Response to Radiation Therapy

The fractionated treatment model is an extension of our previous single-dose radiation model described in (10) that models cell response as a function of early cell death (corresponding to apoptosis) and late cell death (corresponding to mitotic catastrophe). We present the salient details of our single-dose radiation model, but note that the complete development and underlying assumptions are detailed in (10).

#### 2.2.1 Single Species Model of Cell Growth in the Absence of Radiation Therapy

For glioma cell proliferation in the absence of radiation therapy, we augment exponential growth by incorporation of the logistic growth and Allee effect:


(2) $$\frac{dN(t)}{dt}=k_{p}\cdot N(t)\cdot \underset{Allee\;effect}{\underset{\text{ \textbackslash{}underbrace\textbackslash{}, }}{\left(\frac{N(t)}{\theta }+A\right)}}\cdot \underset{logistic\;growth}{\underset{\text{ \textbackslash{}underbrace\textbackslash{}, }}{\left(1-\frac{N(t)}{\theta }\right)}}$$

where *N*(*t*) is the tumor cell confluence, *k_p_* is the proliferation rate (see **Table 1** for a listing of all model parameters, their definition, and units), *A* quantifies the strength of the Allee effect, and *θ* is the carrying capacity (i.e., the maximum number of cells that can fit within a given volume due to space and nutrient limitations). The Allee effect describes the cooperation effects in cell proliferation rate and is proved significant in glioblastoma progression by Neufeld et al. (30). Our previous analysis (10) used model selection to establish that both logistic growth and the Allee effect are necessary to accurately describe our glioma data.

**Table 1:** Model parameters and variables.

Parameter	Unit	Interpretation	Source
***k_p_***	hr^-1^	Proliferation rate	Computed from control, untreated group and fixed throughout experiments
***θ***	1	Carrying capacity	
***A***	1	Allee effect	
***N_p_***	1	Confluence of proliferating cells	Initial cell confluence and total confluence (i.e., *N_p_+N_s_*) are measured from microscopy data.
***N_s_***	1	Confluence of senescent cells	
***N_0_***	1	Initial confluence of cells at time = 0	
***f_DSB_***	1	The fraction of DSBs remaining unrepaired (normalized between 0 and 1)	Measured by flow cytometry
***k_acute,N_***	hr^-1^	Death rate quantifying the contribution of initial confluence to early death	Fit globally with all treated cell response curves.
***a_accum,N_***	1	Scale factor quantifying the contribution of initial confluence to late death	
***k_accum,D_***	hr^-1^	Death rate quantifying the contribution of radiation doses to late death	
***r***	hr^-1^	Radiation efficacy	
***k_ps_***	hr^-1^	Conversion rate from proliferation to senescent components.	

The unit “1” means the parameter is unitless.

#### 2.2.2 Single-Species Model of Cell Growth in the Response to Radiation Therapy

**Figure 2:**
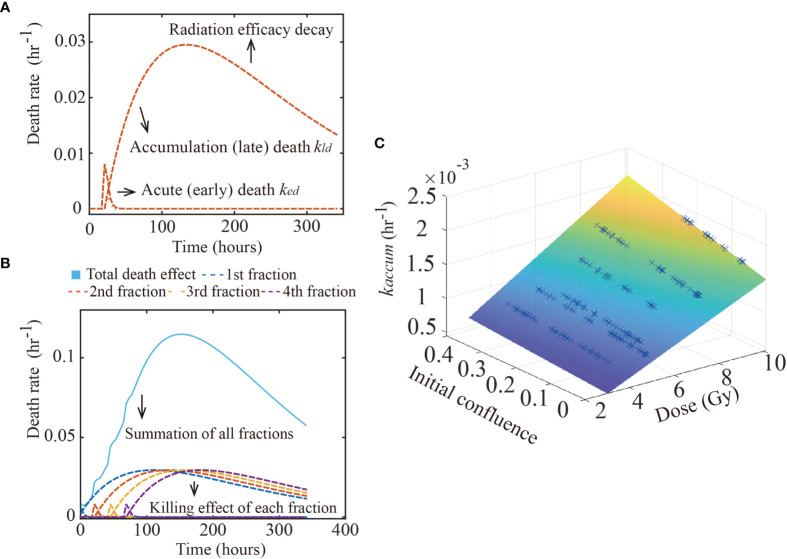
Glioma death rate over time. The figure shows the calibrated early and late death parameters of 9L cell line. Panel **(A)** shows how the early and late death rates change as a function of time from a single treatment. After radiation, the acute death spikes and decreases as the double strand breaks are repaired. In contrast, misrepaired DSBs accumulate within the cells’ genome, causing the late death rate to increase over time, before it eventually decreases as the radiation efficacy decays. Panel **(B)** shows the summation (labeled by the blue solid line) of four fractions (each fraction labeled as dashed lines) irradiated every 24 hours starting from time 0. Each fraction has the same effect as in panel **(A)**. Panel **(C)** illustrates the hypothesis captured by Eq. (7); namely, that *k_accum_* (*D*,*N_0_*) is a function of both dose and initial confluence. Each blue cross indicates the calibrated result of one replicate of the 9L cell line.

After radiation, a small number of cells undergo early apoptosis, which is an outcome of activation of DNA protein kinase and p53 due to excessive DSBs (31); these events occur on the timescale of hours to days (32). Meanwhile, DNA misrepair does not directly kill cells and can accumulate within cells’ genome, eventually triggering chromosome aberration and mitotic catastrophe (33, 34); these events occur on the timescale of days to weeks (32). Based on the above mechanisms, we add early and late death terms to Eq. (2):


(3) $$\frac{dN(t)}{dt}=(k_{p}-k_{ld}(t,D,N_{0}))\cdot \underset{Allee\;effect}{\underset{\text{ \textbackslash{}underbrace\textbackslash{}, }}{\left(\frac{N(t)}{\theta }+A\right)}}\cdot N(t)\cdot \underset{logistic\;growth}{\underset{\text{ \textbackslash{}underbrace\textbackslash{}, }}{\left(1-\frac{N(t)}{\theta }\right)}}-k_{ed}(t,D,N_{0})\cdot N(t)$$

where *k_ed_* and *k_ld_* (both in units of hr^-1^) represent early death and late death rates, respectively, and are a function of time *t*, dose per fraction *D*, and the initial confluency *N_0_*. We use the following equation to describe early cell death as a function of the fraction of unrepaired DSBs within the cell’s genome, *f_DSB_* (*t*):


(4) $$k_{ed}(t,D,N_{0})=\sum_{i=1}^{{}_{number}^{fraction}}k_{accute}(D,N_{0})\cdot f_{DSB}(t^{i},D)$$

(5) $$t^{i}=\{\begin{array}{c} t-24\cdot (i-1)\\ 0 \end{array}\;\;\;\;\;\begin{array}{c} t>24\cdot (i-1)\\ t<24\cdot (i-1) \end{array}$$

where *k_acute_* (*D*,*N_0_*) is the acute death rate. *t^i^* (units: hours) is the time that has passed since the *i* ^th^ fraction. As it would be extremely complex to model the death due to each fraction separately, *k_acute_* (*D*,*N_0_*) is viewed as an average death rate across all fractions and written as:


(6) $$k_{acute}(D,N_{0})=(\alpha_{acute,N}\cdot N_{0}+1)\cdot k_{acute,D}\cdot D$$

where *α_acute_*,*_N_* is a scale factor representing the contribution of acute death due to the initial seeding density, *N_0_*. Biologically, *N_0_* influences (due to cell-cell contact) the proportion of actively proliferating cells at the time of radiation, which determines radiation sensitivity[(as late G2 and M phase is the most sensitive cell cycle (35)]. *k_acute_*,*_D_* is a death rate indicating the contribution of radiation dose to acute death, as larger doses can translate to a higher number of DSBs and, therefore, early apoptosis. **Figure 2** illustrates the changes in *k_ed_* over time. We note that the “+1” in Eq. (6) is for mathematical convenience. When *α_acute_*,*_N_* (i.e., the contribution of the initial seeding density to acute death) is close to 0, Eq. (6) simplifies to *k_acute_* (*D*,*N_0_*)= *k_acute_*,*_D_* ·*D*. Thus, without the “+1”, when *α_acute_*,*_N_* approaches 0, *k_acute_* (*D*,*N_0_*) will also decrease to 0 and the effect of dose will only be determined by seeding density effects.

The “late death” component models mitotic catastrophe of misrepaired cells that occurs following several cell divisions. This population has previously also been referred to as the “abortive fraction” (36). Some cells can survive hours to weeks after radiation before mitotic catastrophe occurs. Here, we model late death as:


(7) $$k_{id}(t,D,N_{0})=\sum_{1}^{{}_{number}^{fraction}}k_{accum}(D,N_{0})\cdot t^{i}\cdot e^{-r\cdot t^{i}}$$

where *k_accum_* (*D*,*N_0_*) is that rate at which the misrepair error first accumulates within the cells’ genome, and *r* (in units of hr^-1^) controls the decay rate of the radiation efficacy. The decay rate of radiation efficacy is viewed as an intrinsic property of each cell line and is a constant across different treatment conditions. Just as for the early death term, we model the parameter *k_accum_* (*D*,*N_0_*) as an average across fractions, instead of modeling each fraction separately. The accumulation death rate *k_accum_* (*D*,*N_0_*) is thus written as:


(8) $$k_{accum}(D,N_{0})=(\alpha_{accum,N}\cdot N_{0}+1)\cdot k_{accum,D}\cdot D$$

where* α_accum_*,*_N_* is a scale factor representing the contribution of late accumulation cell death due to the initial seeding density, *N_0_*. Eq. (8) accounts for the fact that cell density directly impacts the proliferation rate *via* (for example) the proportion cells that are in M phase, which eventually determines how many cells can undergo mitotic catastrophe as it occurs during M phase. *k_accum,D_* is a death rate describing the contribution of radiation dose to accumulation death, as high doses induce a high probability of misrepair [caused by misjoining of clustered DSBs (37)]. Note that Eqs. (6) – (8) have a modified form from our previous single dose study (10); we return to this point in Discussion section.

#### 2.2.3 Two-Species Model of Cell Growth in Response to Radiation Therapy

We construct a two-species model of response to radiation therapy by considering both proliferative and senescent tumor cells. Several mechanisms in radiobiology contribute to the appearance of a senescent population after radiation (38) including (for example) cell cycle checkpoint pathways (39). These senescent cells can remain metabolically active, but undergo irreversible cell cycle arrest and thus can no longer replicate. Without this component, the model assumes cells either return to proliferating (overestimate cell survival) or undergo early or late death (overestimate radiation cell killing), thereby causing a systematic error in the predicted confluence. Therefore, we extended Eq. (3) to include a proliferating compartment, *N_p_* (*t*), and a senescent compartment, *N_s_* (*t*):


(9) $$\frac{dN_{p}(t)}{dt}=(k_{p}-k_{ld}(t,D,N_{0}))\cdot \left(\frac{N_{p}(t)+N_{s}(t)}{\theta }+A\right)\cdot N(t)\cdot \left(1-\frac{N_{p}(t)+N_{s}(t)}{\theta }\right)-k_{ed}(t,D,N_{0})\cdot N_{p}(t)-k_{ps}(D_{total})\cdot N_{0}\cdot N_{p}(t)$$

(10) $$\frac{dN_{s}(t)}{dt}=k_{ps}(D_{total})\cdot N_{0}\cdot N_{p}(t)$$

Eq. (9) - (10) characterize the conversion from the proliferation to senescent compartment due to radiation. As cell-cell contact promotes senescence, we assume that the convertion rate follows a simple linear relation proportional to the initial cell density with the proportionality constant *k_ps_* (units of hr^-1^). Note that the carrying capacity, *θ*, is now shared by both *N_p_* and *N_s_*. Additionally, the conversion rate *k_ps_* is a function of the total dose (i.e., 16 Gy or 20 Gy in our experiments). This simplification (i.e., making *k_ps_* a constant based on the total dose) is because we do not have a direct measurement of the senescent population as this population is changing with each dosing scheme and over time. That is, *k_ps_* should really be a function of time, dose per fraction, and fraction number. To practically realize such an explicit expression for *k_ps_* would require additional experimental measurements.

### 2.3 Numerical Implementation of the Mathematical Models

ODE models were implemented *via* the finite difference method with a fully explicit forward Euler formulation with a time step of 0.01 hrs with initial condition, *N_p_* (0), equal to the cell confluence measurements at time 0 and *N_s_* (0) = 0. Early acute death rate term is multiplied by a smooth heaviside step function, *via* the hyperbolic tangent function; tanh [*f_DSB_* (*t*)], to prevent curve discontinuity.

### 2.4 Model Selection

Eqs. (1) – (10) are based on general radiobiology mechanisms. For specific cell lines with different signaling pathways and radiation sensitivity, it is unclear if this model is appropriate to describe the data. Therefore, it is crucial to perform model selection prior to applying the model to a specific cell line. Starting from the above “full model”, we systematically remove one or two parameters, yielding seven competing “daughter” models. Using the early death term as an example, the initial seeding density *N_0_* and doses *D* might not have an impact on early apoptosis for the 9L and C6 cell lines. By removing either *α_acute_*,*_N_* or *k_accum,D_* or both, we obtain three “daughter” models (i.e., models 2-4 in section 2 of the **Supplementary Materials**). Similarly, removing the late death term (three models) or the senescent term (one model) yields an additional seven “daughter” models. (See the section 2 of the **Supplementary Materials** for the formulation of all eight models.). For each model, parameters are fit with the Levenberg-Marquardt algorithm (*via* “lsqnonlin” in MATLAB). To determine which mechanisms are required for optimally characterizing 9L and C6 data and to obtain the most parsimonious model, we perform model selection on these eight models *via* the Akaike information criterion (AIC) (40):


(11) $$AIC=n\cdot ln\frac{RSS}{n}+2p+\frac{2p^{2}+2p}{n-p-1}$$

where *n* is the sample number (i.e., the number of cell confluency curves in our scenario), RSS is the residual squared sum between the model fit and data, and *p* is the number of free parameters. The AIC finds the most parsimonious model by balancing the relative goodness-of-fit with the number of free parameters. Specifically, we build our training set by randomly picking seventy-five percent of the data under each treatment condition, leading to 262 replicates for the 9L cells and 211 replicates for the C6 cells. (That is, we pick 75% from four fractions of 4 Gy, 75% from two fractions of 10 Gy, etc., thereby ensuring that each treatment condition is equally represented in the training set.) The remaining 25% of the data is used for validation. (Note that the word ‘replicate’ is in reference to our experiments; that is, we repeated a group of independent wells with the same treatment schedules at time point 0. The response from each well over the 340 hours won’t be identical as they are affected by (for example) subtle variations in the cells’ phenotype or genotype, variations in initial seeding density, etc.)

The AIC-based model selection is then performed on all eight models including the full model using the training set. By globally fitting the training set (i.e., 262 9L replicates and 211 C6 replicates, to these eight models respectively) we obtain the residual squared sum over the entire training set for each model. The model that returns the lowest AIC score is selected as the most parsimonious. We also compute the Akaike weights *via*:


(12) $$\omega_{i}=\frac{\exp(\delta_{i})}{\sum_{j=1}^{8}\exp(\delta_{i})}$$

(13) $$\delta_{i}=\frac{AIC_{i}-AIC_{\min}}{AIC_{\min}}$$

where *AIC_i_* is the AIC score of the *i* ^th^ model, and *AIC_min_* is the minimum AIC observed among all eight models. These weights are used to compare the models to each other.

### 2.5 Parameter Calibration

**Table 1** lists parameter definitions and the methods we use to obtain these parameters. We previously (10) fit untreated cell data to Eq. (1) to obtain the proliferation rate, *k_p_*, carrying capacity, *θ*, and the Allee constant, *A*, for both the 9L and C6 cells. These parameters are assumed constant throughout the present study. The standard deviation of the estimated model parameters is computed *via* “nlparci” in MATLAB, and employed to generate a corresponding distribution *via* “makedist”. As the biological definitions of these parameters specify their values must be positive, the parameters are given a lower bound of zero during calibration. Note that during model calibration, we fit parameters globally in the sense that all curves from the training set, consisting of both the 16 Gy and 20 Gy total dose groups, are fit together since all parameters are independent of dose and initial cell density except for the conversion rate *k_ps_*. As *k_ps_* is a function of the total dose, we treat *k_ps_* at 16 Gy and at 20 Gy as two individual parameters in our calibration process. Distributions of the calibrated parameters are compared between the two cell lines to verify if they are significantly different by the z-test (*via* “ztest” in MATLAB at the 5% significance level).

### 2.6 Model Validation and Error Analysis

Validation is performed on the AIC selected model using the remaining twenty-five percent of data (i.e., 91 9L curves and 74 C6 curves), which are “unseen” during both the model selection and parameter calibration steps. The forward model has two inputs: initial seeding density (*N_0_*) and dose schedule. We run the model forward using the calibrated parameters as shown in **Figure 5**. The prediction mean and intervals are then computed *via* the MATLAB function “nlpredci” by inputting the calibrated parameters, Jacobian matrix, and residuals determined by the “lsqnonlin” during calibration. Radiation responses are predicted using the same set of parameters regardless of treatment dose schedules or initial density. For example, predicting the effects of the four fractions of 4 Gy on the low confluence group employs the same set of parameters as predicting the effects of the two fractions of 10 Gy on the high confluence group (except for the *k_ps_*, which is a constant based on total dose). To quantify the predictive accuracy, we compare the measured and model prediction means using the Pearson correlation coefficient (PCC) and concordance correlation coefficient (CCC).

## 3 Results

### 3.1 Image Segmentation and Cell Response Curves

**Figure 3:**
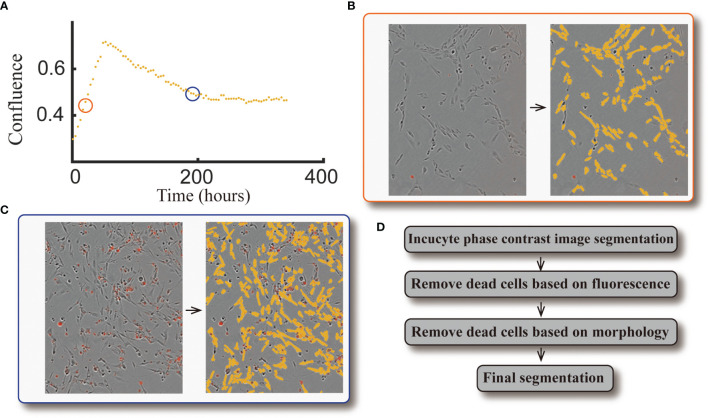
Example of cell response curve and image segmentation. Panel **(A)** shows the cell response curve from one replicate of the 9L cell line treated with four fractions of 4 Gy. The left portion of Panel **(B)** is the raw data at 40 hours [indicated by the orange circle in panel **(A)**] obtained *via* live cell microscopy. The image is presented as phase-contrast with a red fluorescent label indicating dead cells. The corresponding segmentation (highlighted by yellow) is on the right portion of panel **(B)**. The left portion of Panel **(c)** is the raw data at 190 hours [indicated by the blue circle in panel **(A)**], where there are a large number of dead cells compared to the early time point. The corresponding segmentation is presented in the right portion of the panel. Panel **(D)** briefly summarizes our segmentation pipeline; details were provided in ref. (10).

The same image segmentation pipeline from our previous study (10) is used here. When comparing the automatically segmented images to our manually segmented baseline, this segmentation pipeline achieves an average Sørensen–Dice coefficient of 0.79 (i.e., 21% error). See **Figure 3** for an example of a cell response curve that received four fractions of 4 Gy and its corresponding image segmentation.

### 3.2 Model Selection

**Figure 4:**
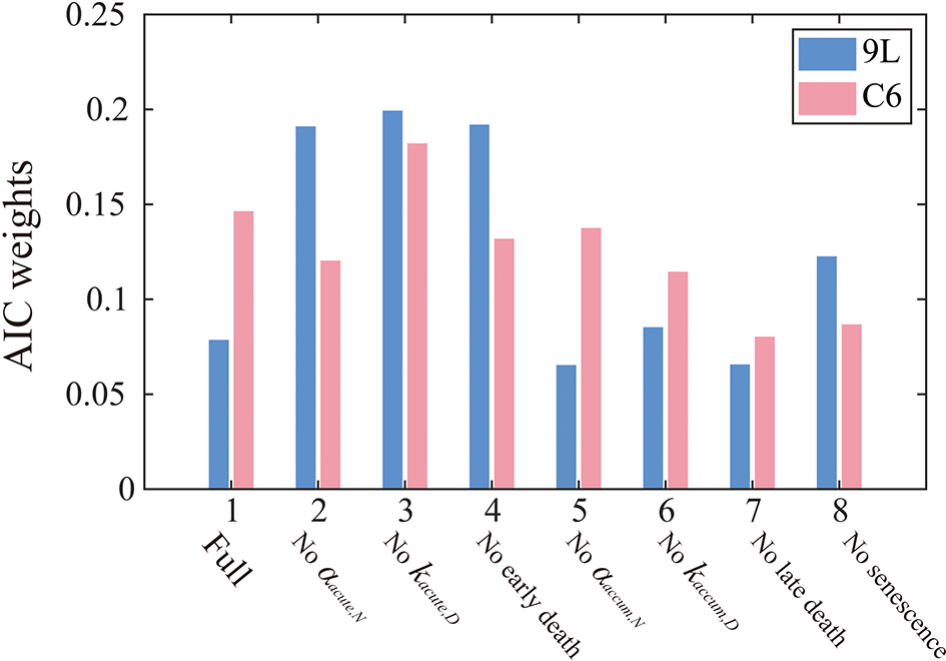
AIC weights for each model models. The label, “Full” on the horizontal axis indicates that all model components are included [i.e., Eq. (1) – (10)], while the other labels indicate what portion of the Full model was removed in that particular reduced model. AIC weights can be interpreted as the probability that a particular model is preferred for modeling the 9L (blue) or C6 (red) cell line. In particular, Model 3 is most frequently selected by the AIC for both the 9L and C6 cell lines. Models without accumulation effects (i.e., model 5-8) generally perform worse than models without early effects (model 2-4), indicating the importance of incorporating the effects of late cell killing over acute apoptosis.

**Figure 4** summarizes the AIC weights across all eight models. As it has the highest weights for both the 9L and C6 cell lines, Model 3 was selected as the most parsimonious model from the training set and thus will be used for parameter calibration and prediction. While the complete formulation is presented in the section 2 of the **Supplementary Materials**, Model 3 combines early apoptosis (*k_acute,N_*), mitotic catastrophe (*α_accum,N_*, *k_accum,D_*, *r*), and senescence [*k_ps_* (16 Gy), *k_ps_* (20 Gy)] as given by the following system of equations:


(14) $$\frac{dN_{p}(t)}{dt}=(k_{p}-k_{ld}(t,D,N_{0}))\cdot \left(\frac{N_{p}(t)+N_{s}(t)}{\theta }+A\right)\cdot N(t)\cdot \left(1-\frac{N_{p}(t)+N_{s}(t)}{\theta }\right)\;-k_{ed}(t,D,N_{0})\cdot N_{p}(t)-k_{ps}(D_{total})\cdot N_{0}\cdot N_{p}(t)$$

(15) $$k_{ed}(t,D,N_{0})=\sum_{i=1}^{{}_{number}^{fraction}}k_{accute,N}\cdot N_{0}\cdot f_{DSB}(t^{i},D)$$

(16) $$k_{id}(t,D,N_{0})=\sum_{1}^{{}_{number}^{fraction}}(\alpha_{accum,N}\cdot N_{0}+1)\cdot k_{accum,D}\cdot t^{i}\cdot e^{-r\cdot t^{i}}$$

(17) $$\frac{dN_{s}(t)}{dt}=k_{ps}(D_{total})\cdot N_{0}\cdot N_{p}(t)$$

Note that the parameter *k_acute_*,*_D_* in Eq. (6) is removed and the corresponding expression for early death is rewritten as in Eq. (15). This was done since model selection indicated that *k_acute_*,*_D_* is not required to characterize our data. Consequently, we removed *k_acute_*,*_D_* and modified the notation of *α_acute_*,*_N_* in Eq. (6) to *k_acute_*,*_N_* (in units of hr^-1^) in Eq. (15) to represent the death rate.

From the AIC score, model 3 [i.e., Eqs. (14) – (17)] is 1.18 times more likely to be the best model than model 4 ‘no early death’ model, 2.61 times more likely than model 7 ‘no late death model’, and 1.82 times more likely than model 8 ‘no senescence’ single species model. These results indicate the importance of accumulation effects (i.e., accumulating DNA misrepair, mitotic catastrophe and gradual conversion to senescence) over acute effects (i.e., early apoptosis) to quantify the time courses of 9L and C6 radiation response. Note that the goal of the model selection process is to identify the most parsimonious model to describe our time-resolved data, rather than the model that includes the most biology. As for the 9L and C6 cell lines, most cells die due to late mitotic catastrophe while early apoptosis only kills a minority of cells. Thus, removing the radiation dose effect from the early death term as in model 3 does not harm the model’s ability to characterize the data.

### 3.3 Parameter Calibration

**Figure 5:**
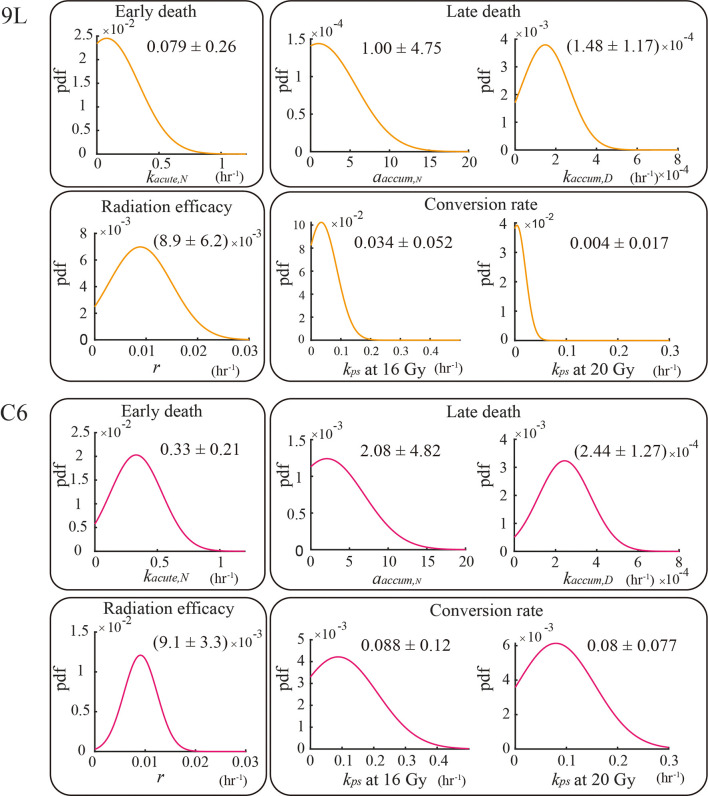
Parameter calibration results. All parameters are fit globally using the training set and are independent of initial seeding density or dose schedules (e.g., the four fractions of 4 Gy curves share the same set of parameters as the two fractions of 10 Gy curves for both the 9L and C6; the one exception is for *k_ps_*, where we treat *k_ps_* (16 Gy) and *k_ps_* (20 Gy) as two individual parameters. The biological interpretation of the parameters requires that these parameters must take on a value great than or equal to 0; thus, the lower bound of the parameters is set to zero during calibration.

The AIC selected model has six parameters *k_acute,N_*, *α_accum,N_*, *k_accum,D_*, *r*, *k_ps_* (16 Gy), and *k_ps_* (20 Gy). The top panel in **Figure 5** shows the calibrated parameters for the 9L cells, while the bottom panel shows the calibrated parameters for the C6 cells. Note that the model allows to quantify the degree to which the C6 cell line is more radiation sensitive than the 9L cell line, as has been previously reported (41). In particular, the early death rates *k_acute,N_* (p value = 9.5e-23), late death rates *k_accum,D_* (p value = 8.1e-21) and conversion rate to senescent component *k_ps_* (p value = 1.6e-20 for 16 Gy and 0.0 for 20 Gy) of the C6 are all significantly larger than 9L *via* the z-test. Both the 9L and C6 exhibit a similar radiation efficacy decay *r* (p value = 0.44, i.e., no significant difference *via* z-test), suggesting a similar duration of radiation cell killing persisting on both cell lines. The proliferation rate, carrying capacity, and Allee effect parameter values, as well as the number of training curves for calibration, are provided in the section 1 of the **Supplementary Material**.

### 3.4 Model Validation and Error Analysis

**Figure 6:**
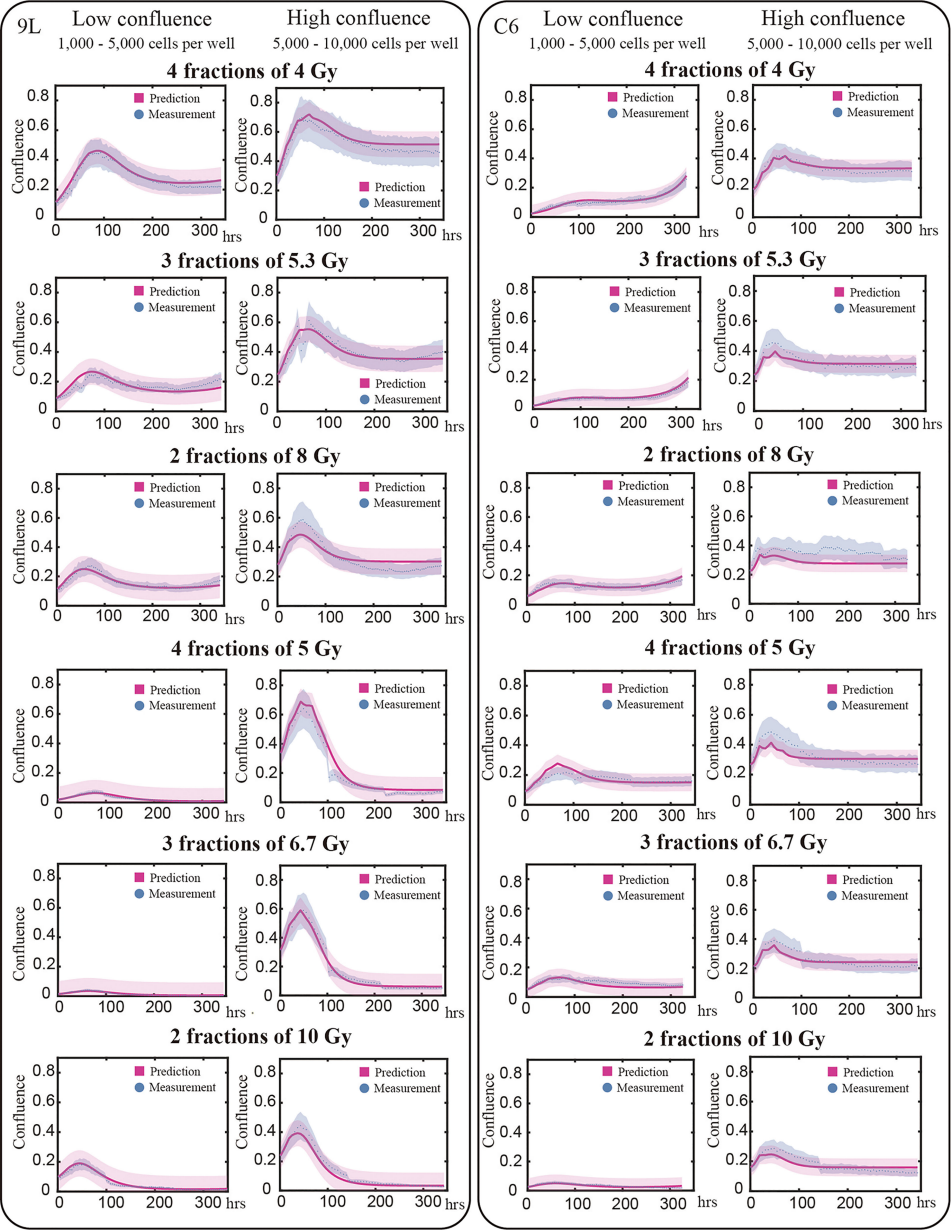
Model validation. The prediction interval (labeled by red) and measured microscopy data (labeled blue dots) are plot for representative examples of each initial and treatment condition. Previously we determined our average segmentation error was 21% using the Sørensen–Dice coefficient (10). This segmentation error is indicated by the blue intervals. Each row in this figure shows different treatment schedules labeled on the left; for example, the first row shows cells treated with four fractions of 4 Gy. Each column stands for the initial confluence for a specific cell line; for example, the first column represents low initial seeding density for 9L cell line. Predictions are made globally for each of the cell lines; that is, predictions for the 9L cells with an initial low confluence receiving four fractions of 4 Gy are made using the same set of parameters as the 9L cells with an initial high confluence receiving two fractions of 10 Gy. Given the initial confluence and treatment schedule as the two inputs, our model makes accurate predictions across a wide range of initial conditions. However, the model is not perfect as the prediction typically undershoots the first peak while overshooting the tail for C6 cell line; an important point we return to in the Discussion section.

We use the validation group (25% of our total data, 91 9L curves and 74 C6 curves) to evaluate the predictive accuracy of our model. **Figure 6** presents examples of the model validation results with each column representing one initial confluence of a specific cell line (e.g., the first column shows a low initial seeding confluence of the 9L cell line). Each row shows a different fractionation schedule (e.g., the first row shows cells receiving four fractions of 4 Gy radiation). The error bar on the measurement (labeled by blue) is based on the image segmentation error (21%) from our previous study (10), as the same segmentation pipeline is used. The prediction error (labeled by red) is computed from MATLAB function ‘nlpredci’, which computes the prediction interval *via* the Delta method based on the Jacobian matrix.

For the 9L group receiving a total dose of 16 Gy, the average PCC and CCC between the predicted and the measured data are 0.92 ± 0.009 (average ± standard error) and 0.78 ± 0.014, respectively. For the 9L group receiving a total of 20 Gy, the PCC and CCC are 0.98 ± 0.001 and 0.96 ± 0.002, respectively. For the C6 group receiving a total of 16 Gy, the PCC and CCC are 0.89 ± 0.011 and 0.77 ± 0.020, respectively. Finally, for the C6 group receiving a total of 20 Gy, the PCC and CCC are 0.90 ± 0.004 and 0.73 ± 0.014, respectively. Details of PCC and CCC values for each treatment condition is provided in **Table 2**. Overall, the accuracy of prediction is superior for the 9L cell line than the C6 cell line. This is at least partially due to the heterogeneous radiation response observed across the C6 replicates; we return to this important point in Discussion section.

**Table 2:** Error between model prediction and measurement.

	Treatment schedule	9L		C6	
		PCC	CCC	PCC	CCC
**16 Gy**	4 fractions of 4 Gy	0.96 ± 0.003	0.84 ± 0.011	0.91 ± 0.007	0.85 ± 0.012
	3 fractions of 5.3 Gy	0.85 ± 0.015	0.71 ± 0.019	0.89 ± 0.008	0.76 ± 0.014
	2 fractions of 8 Gy	0.96 ± 0.001	0.79 ± 0.003	0.84 ± 0.016	0.65 ± 0.029
**20 Gy**	4 fractions of 5 Gy	0.97 ± 0.002	0.96 ± 0.002	0.90 ± 0.005	0.76 ± 0.011
	3 fractions of 6.7 Gy	0.98 ± 0.001	0.93 ± 0.001	0.88 ± 0.004	0.64 ± 0.016
	2 fractions of 10 Gy	0.98 ± 0.001	0.95 ± 0.003	0.91 ± 0.004	0.77 ± 0.011

Mean ± standard error.

## 4 Discussion

We have proposed an experimental-computational system that includes a mathematical model that characterizes the dynamic cell response of the 9L and C6 glioma receiving fractionated radiation. Model parameters are calibrated using a training set of various initial seeding densities and dose schedules over the course of two weeks. Next, the calibrated parameters are applied to a validation set to predict response to radiation therapy. Each term in our model has an explicit or implicit (i.e., *α_accum,N_*, *k_ps_* (16 Gy), *k_ps_* (20 Gy) are indirectly related to cell cycles) biological definition. For example, early apoptosis and late mitotic catastrophe are captured by the parameters *k_acute,N_* and *k_accum,D_*, *r*, respectively. This approach has several advantages over current models as it explicitly includes the temporal dynamics of several biological mechanisms related to the response of cells to fractionated radiation.

Mathematically, the LQ model describes the fraction of survival cells at experimental endpoints given a specific radiation dose, and therefore is not designed for calibrating with time-resolved data. Though there are mathematical models that account for temporal dynamics by embedding the LQ term into the death rates (42), the true death rates are unlikely to obey the LQ relation throughout the whole time course as it ignores time dependent phenomena such as early apoptosis and late mitotic catastrophe. Time-dependent radiobiologic mechanisms are often ignored in these modeling studies, a limitation possibly due to the historical difficulty of accessing radiation response data with high temporal resolution. However, recent advances in imaging techniques can now provide the requisite, high temporal resolution data (43–45) appropriate for model calibration. When we mathematically characterize these time series data, a dynamic model is required, since the measurement interval (e.g., hours) is much shorter than the length of the experiment when the surviving fraction would be determined *via* the LQ model. To the best of our knowledge, this study is the first mechanism-based fractionated radiation model verified by the cell experimental data at high temporal-resolution. We have constructed this approach by building on previous time-resolved dynamic radiation models that either calibrated to a single dose of radiation (46), or to data collected every several days (23) or weeks (47). We hope that the current effort can provide motivation for focusing on factors that describe the dynamics of radiobiology as a function of time, thereby potentially providing a new way to guide and optimize radiation dose scheduling.

As each term in the selected model system [i.e., Eqs. (14) – (17)] is based on an underlying mechanism, the calibrated parameters summarized in **Figure 5** are easily interpreted in light of the underlying biology. Though the specific values of parameters may differ for each cell line (a not unexpected observation), the trend observed (i.e., radiosensitive cells exhibit significantly larger death rates) may carry over to other cell lines since the proposed model and the parameters are based on biological mechanisms common to a wide range of cell lines (48). Thus, the model summarized by Eqs. (14) – (17) is likely applicable to other cell lines for which radiation treatment is of interest, thereby having a significant impact beyond gliomas.

Despite the advantages, there are several opportunities for improvement in both the experimental and modeling components of the study. For example, note that in **Figure 6** some curves suffer a discontinuous jump (e.g., at approximately 110 hours in the “4 fractions of 5 Gy” high confluence group). This is due to the cell loss when we refresh the media. While we have implemented a method to change the media as delicately as possible (as described in section 2.1.2), we are still aspirating an unknown number of live cells at each media change. Potentially more significant, though, is how we handle the senescent component. Since we did not have a method in place that allows us to directly measure this component over time, we are forced to infer its existence and behavior *via* parameter calibration. Clearly, incorporating longitudinal measurements of the fractions of senescent cells is required to generalize the model to other dosing schemes.

Areas for improvement on the modeling side include developing a more rigorous linking between radiation dose and the rate constants. For example, note that Eqs. (6) and (8) have a different form from our previous single-dose study (10). The main reason for the changes is due to the dose range in this study (4 - 10 Gy per fraction) compared to the previous study (a single fraction of 2 – 16 Gy). In our previous single-fraction study we observed a saturation effect in the death rates; i.e., the death rates do not significantly increase above a certain dose threshold. This is because cell death does not happen instantaneously; i.e., the cell death pathways require time to be executed. Thus, we use Michaelis–Menten equations to characterize the observed saturation effect. However, in the present study, we use a narrower range of radiation dose and we did not observe the saturation in cell killing within this dose range. Thus, we use a simple linear relation for the acute death, *k_acute_* (*D*,*N_0_*), accumulation death, *k_accum_* (*D*,*N_0_*), and a constant radiation efficacy *r*, versus radiation doses. Consequently, the death rates *k_acute_* (*D*,*N_0_*), *k_accum_* (*D*,*N_0_*), and *r* most likely require future modifications when applied to dose schedules involving larger doses. We also note that, compared to the previous single-dose study [i.e., Eq. (6) in (10)], we removed the parameter *T* in Eq. (7). This parameter assumes there is a time delay, *T*, before the accumulation of misrepairs can start. Our previous study established its value between the first 20-40 hours after radiation for the 9L cell line, and 0 hours for the C6 cell line [consistent with the established radiosensitivity of the C6 cell line (41)]. In current fractionated dosing scheme, as we extend the experiment time to approximately 340 hours, the value of *T* is much less than the length of the experiment. Thus, it is reasonable to remove the parameter *T* for simplicity. Another limitation is that Eqs. (14) – (17) do not (of course) account for all of the most prominent radiobiological processes. For example, we assume a fixed conversion rate between the proliferative and senescent components—a parameter that is likely to change with time, dose per fraction, and fraction numbers, and therefore would require a function of its own to describe its temporal dynamics. Indeed, this may explain the undershoot peak or overshoot tail in **Figure 6**. A second area for improvement in Eqs. (14) – (17) is the explicit incorporation of phenotypic or genomic heterogeneity across the population, which almost certainly affects the early (*k_acute,N_*) and late (*k_accum,D_*) death effects between different replicates. This explains why the correlation coefficients in **Table 2** indicate the model performs worse under certain dose schedules for the C6 cell line. See section 3 of the **Supplementary Materials** for an example of the heterogeneous response we observe in two replicates of C6 cells treated with the same three fractions of 6.7 Gy (note this is the group with the lowest PCC and CCC value). Accounting for such heterogeneity to improve the predictive accuracy of the model will be the focus of future study. As presented, the current model has limited clinical application because it is formulated for describing the 2D dynamics of cells in a dish which is, of course, very different than the *in vivo* situation. However, the concept of using time-resolved data to calibrate a biologically-based, mathematical model to make patient specific predictions is highly translatable and, indeed, something we have investigated at length in both the *in vivo* pre-clinical (49–53) and clinical (24, 54–58) settings.

## 5 Conclusion

We have extended our previous single-dose model to account fractionated treatment, and successfully validated the resulting model with *in vitro* experimental microscopy data. This study demonstrates a promising experimental-mathematical approach based on radiobiology mechanisms that can accurately predict the temporal dynamics of the response of glioma cells to radiation. Future efforts include linking the model to our tissue scale formalism for predicting response in patients (23), and employing the methods of optimal control theory to optimize treatment outcomes in pre-clinical murine studies (59).

## Data Availability Statement

The original contributions presented in the study are included in the article/**Supplementary Material**. Further inquiries can be directed to the corresponding author.

## Author Contributions

JL collected data, performed modeling analysis and wrote the manuscript. JY helped with the experiments. DH and TY conceptualized and supervised the study, reviewed and edited the manuscript. All authors have read and approved the final manuscript.

## Funding

This work is supported by the National Cancer Institute *via* R01CA240589, R01 CA235800, U01CA253540, and R01CA186193, and the Cancer Prevention and Research Institute of Texas (CPRIT) *via* RR160005. TY is a CPRIT Scholar of Cancer Research.

## Conflict of Interest

The authors declare that the research was conducted in the absence of any commercial or financial relationships that could be construed as a potential conflict of interest.

## Publisher’s Note

All claims expressed in this article are solely those of the authors and do not necessarily represent those of their affiliated organizations, or those of the publisher, the editors and the reviewers. Any product that may be evaluated in this article, or claim that may be made by its manufacturer, is not guaranteed or endorsed by the publisher.
