# Supplementary material for: A Multi-Compartment Model of Glioma Response to Fractionated Radiation Therapy Parameterized via Time-Resolved Microscopy Data
Source: Front Oncol. 2022 Feb 4;12:811415. doi: 10.3389/fonc.2022.811415 (PMC8855115; doi:10.3389/fonc.2022.811415)
Supplement: Supplementary file 1 [file DataSheet_1.docx]

**Supplementary materials: A multi-compartment model of glioma response to fractionated radiation therapy parameterized *via* time-resolved microscopy data**

^1^Junyan Liu, ^4,5^David A. Hormuth II, ^1^Jianchen Yang, ^1-6^Thomas E. Yankeelov*

Departments of ^1^Biomedical Engineering, ^2^Diagnostic Medicine, ^3^Oncology, ^4^Oden Institute for Computational Engineering and Sciences, ^5^Livestrong Cancer Institutes

The University of Texas at Austin

Austin, Texas 78712

^6^Department of Imaging Physics

The University of Texas MD Anderson Cancer Center

Houston, Texas, 77030

Supplementary materials

**S1. Parameter calibration**

The proliferation rate, *k_p_*, carrying capacity, *θ*, and Allee effect, *A*, are calibrated from untreated cells, and are used as constants throughout the study. The 9L cell line has an average cell doubling time of 26 hours, with *k_p_* = 0.079 hr^-1^, *θ* = 0.98, and *A* = 0.33. The C6 cell line has an average cell doubling time of 18 hours, with *k_p_* = 0.16 hr^-1^, *θ* = 0.81, and *A* = 0.15. The number of training and validation curves for each dose and cell line are summarized below.

|  | 9L | | C6 | |
| --- | --- | --- | --- | --- |
| Curve numbers | Training | Validation | Training | Validation |
| 4 fractions of 4 Gy | 45 | 15 | 44 | 16 |
| 3 fractions of 5.3 Gy | 40 | 13 | 39 | 14 |
| 2 fractions of 8 Gy | 45 | 15 | 27 | 9 |
| 4 fractions of 5 Gy | 44 | 16 | 21 | 8 |
| 3 fractions of 6.7 Gy | 44 | 16 | 38 | 12 |
| 2 fractions of 10 Gy | 44 | 16 | 42 | 15 |

**S2. Model selection**

Starting from the full model (i.e., Eqs. (1) – (9) in the manuscript), we remove each biological mechanism one by one, yielding seven “daughter” models, as the following:

1. Full model

The full model is defined by Eqs. (S1) – (S4):

| $\begin{matrix} \frac{dN_{p}(t)}{dt}=(k_{p}-k_{ld}(t,D,N_{0}))\cdot\left(\frac{N_{p}(t)+N_{s}(t)}{\theta}+A\right)\cdot N_{p}(t)\cdot\left(1-\frac{N_{p}(t)+N_{s}(t)}{\theta}\right) \\ -k_{ed}(t,D,N_{0})\cdot N_{p}(t)-k_{ps}(D_{total})\cdot N_{0}\cdot N_{p}(t) \end{matrix}$ | (S1) |
| --- | --- |
| $k_{ed}(t,D,N_{0})=\sum_{i=1}^{\begin{matrix} fraction \\ number \end{matrix}} (\alpha_{acute,N}\cdot N_{0}+1)\cdot k_{acute,D}\cdot D\cdot f_{DSB}(t^{i},D)$ | (S2) |
| $k_{ld}(t,D,N_{0})=\sum_{1}^{\begin{matrix} fraction \\ number \end{matrix}} (\alpha_{accum,N}\cdot N_{0}+1)\cdot k_{accum,D}\cdot D\cdot t^{i}\cdot e^{-r\cdot t^{i}}$ | (S3) |
| $\frac{dN_{s}(t)}{dt}=k_{ps}(D_{total})\cdot N_{0}\cdot N_{p}(t)$ | (S4) |

1. Remove *α_acute_*_,_*_N_* , which assumes the seeding density (from early death) has a negligible effect on the model’s ability to characterize the data. This results in the model defined by Eqs. (S5) – (S8):

| $\begin{matrix} \frac{dN_{p}(t)}{dt}=(k_{p}-k_{ld}(t,D,N_{0}))\cdot\left(\frac{N_{p}(t)+N_{s}(t)}{\theta}+A\right)\cdot N_{p}(t)\cdot\left(1-\frac{N_{p}(t)+N_{s}(t)}{\theta}\right) \\ \text{ }-k_{ed}(t,D,N_{0})\cdot N_{p}(t)-k_{ps}(D_{total})\cdot N_{0}\cdot N_{p}(t) \end{matrix}$ | (S5) |
| --- | --- |
| $k_{ed}(t,D,N_{0})=\sum_{i=1}^{\begin{matrix} fraction \\ number \end{matrix}} k_{acute,D}\cdot D\cdot f_{DSB}(t^{i},D)$ | (S6) |
| $k_{ld}(t,D,N_{0})=\sum_{1}^{\begin{matrix} fraction \\ number \end{matrix}} (\alpha_{accum,N}\cdot N_{0}+1)\cdot k_{accum,D}\cdot D\cdot t^{i}\cdot e^{-r\cdot t^{i}}$ | (S7) |
| $\frac{dN_{s}(t)}{dt}=k_{ps}(D_{total})\cdot N_{0}\cdot N_{p}(t)$ | (S8) |

1. Remove *k_acute_*_,_*_D_*, which assumes the radiation dose (from early death) has a negligible effect on the model’s ability to characterize the data. This results in the model defined by Eqs. (S9) – (S12):

| $\begin{matrix} \frac{dN_{p}(t)}{dt}=(k_{p}-k_{ld}(t,D,N_{0}))\cdot\left(\frac{N_{p}(t)+N_{s}(t)}{\theta}+A\right)\cdot N_{p}(t)\cdot\left(1-\frac{N_{p}(t)+N_{s}(t)}{\theta}\right) \\ \text{ }-k_{ed}(t,D,N_{0})\cdot N_{p}(t)-k_{ps}(D_{total})\cdot N_{0}\cdot N_{p}(t) \end{matrix}$ | (S9) |
| --- | --- |
| $k_{ed}(t,D,N_{0})=\sum_{i=1}^{\begin{matrix} fraction \\ number \end{matrix}} k_{acute,N}\cdot N_{0}\cdot f_{DSB}(t^{i},D)$ | (S10) |
| $k_{ld}(t,D,N_{0})=\sum_{1}^{\begin{matrix} fraction \\ number \end{matrix}} (\alpha_{accum,N}\cdot N_{0}+1)\cdot k_{accum,D}\cdot D\cdot t^{i}\cdot e^{-r\cdot t^{i}}$ | (S11) |
| $\frac{dN_{s}(t)}{dt}=k_{ps}(D_{total})\cdot N_{0}\cdot N_{p}(t)$ | (S12) |

1. Remove *k_ed_*, which assumes early death has a negligible effect on the model’s ability to characterize the data. This results in the model defined by Eqs. (S13) – (S15):

| $\begin{matrix} \frac{dN_{p}(t)}{dt}=(k_{p}-k_{ld}(t,D,N_{0}))\cdot\left(\frac{N_{p}(t)+N_{s}(t)}{\theta}+A\right)\cdot N_{p}(t)\cdot\left(1-\frac{N_{p}(t)+N_{s}(t)}{\theta}\right) \\ \text{ }-k_{ps}(D_{total})\cdot N_{0}\cdot N_{p}(t) \end{matrix}$ | (S13) |
| --- | --- |
| $k_{ld}(t,D,N_{0})=\sum_{1}^{\begin{matrix} fraction \\ number \end{matrix}} (\alpha_{accum,N}\cdot N_{0}+1)\cdot k_{accum,D}\cdot D\cdot t^{i}\cdot e^{-r\cdot t^{i}}$ | (S14) |
| $\frac{dN_{s}(t)}{dt}=k_{ps}(D_{total})\cdot N_{0}\cdot N_{p}(t)$ | (S15) |

1. Remove *α_accum_*_,_*_N_*, which assumes the seeding density (from late death) has a negligible effect on the model’s ability to characterize the data. This results in the model defined by Eqs. (S16) – (S19):

| $\begin{matrix} \frac{dN_{p}(t)}{dt}=(k_{p}-k_{ld}(t,D,N_{0}))\cdot\left(\frac{N_{p}(t)+N_{s}(t)}{\theta}+A\right)\cdot N_{p}(t)\cdot\left(1-\frac{N_{p}(t)+N_{s}(t)}{\theta}\right) \\ \text{ }-k_{ed}(t,D,N_{0})\cdot N_{p}(t)-k_{ps}(D_{total})\cdot N_{0}\cdot N_{p}(t) \end{matrix}$ | (S16) |
| --- | --- |
| $k_{ed}(t,D,N_{0})=\sum_{i=1}^{\begin{matrix} fraction \\ number \end{matrix}} (\alpha_{acute,N}\cdot N_{0}+1)\cdot k_{acute,D}\cdot D\cdot f_{DSB}(t^{i},D)$ | (S17) |
| $k_{ld}(t,D,N_{0})=\sum_{1}^{\begin{matrix} fraction \\ number \end{matrix}} k_{accum,D}\cdot D\cdot t^{i}\cdot e^{-r\cdot t^{i}}$ | (S18) |
| $\frac{dN_{s}(t)}{dt}=k_{ps}(D_{total})\cdot N_{0}\cdot N_{p}(t)$ | (S19) |

1. Remove *k_accum,D_*, which assumes the radiation dose (from late death) has a negligible effect on the model’s ability to characterize the data. This results in the model defined by Eqs. (S20) – (S23):

| $\begin{matrix} \frac{dN_{p}(t)}{dt}=(k_{p}-k_{ld}(t,D,N_{0}))\cdot\left(\frac{N_{p}(t)+N_{s}(t)}{\theta}+A\right)\cdot N_{p}(t)\cdot\left(1-\frac{N_{p}(t)+N_{s}(t)}{\theta}\right) \\ \text{ }-k_{ed}(t,D,N_{0})\cdot N_{p}(t)-k_{ps}(D_{total})\cdot N_{0}\cdot N_{p}(t) \end{matrix}$ | (S20) |
| --- | --- |
| $k_{ed}(t,D,N_{0})=\sum_{i=1}^{\begin{matrix} fraction \\ number \end{matrix}} (\alpha_{acute,N}\cdot N_{0}+1)\cdot k_{acute,D}\cdot D\cdot f_{DSB}(t^{i},D)$ | (S21) |
| $k_{ld}(t,D,N_{0})=\sum_{1}^{\begin{matrix} fraction \\ number \end{matrix}} k_{accum,N}\cdot N_{0}\cdot t^{i}\cdot e^{-r\cdot t^{i}}$ | (S22) |
| $\frac{dN_{s}(t)}{dt}=k_{ps}(D_{total})\cdot N_{0}\cdot N_{p}(t)$ | (S23) |

1. Remove *k_ld_*, which assumes the late death has a negligible effect on the model’s ability to characterize the data. This results in the model defined by Eqs. (S24) – (S26):

| $\begin{matrix} \frac{dN_{p}(t)}{dt}=(k_{p})\cdot\left(\frac{N_{p}(t)+N_{s}(t)}{\theta}+A\right)\cdot N_{p}(t)\cdot\left(1-\frac{N_{p}(t)+N_{s}(t)}{\theta}\right) \\ \text{ }-k_{ed}(t,D,N_{0})\cdot N_{p}(t)-k_{ps}(D_{total})\cdot N_{0}\cdot N_{p}(t) \end{matrix}$ | (S24) |
| --- | --- |
| $k_{ed}(t,D,N_{0})=\sum_{i=1}^{\begin{matrix} fraction \\ number \end{matrix}} (\alpha_{acute,N}\cdot N_{0}+1)\cdot k_{acute,D}\cdot D\cdot f_{DSB}(t^{i},D)$ | (S25) |
| $\frac{dN_{s}(t)}{dt}=k_{ps}(D_{total})\cdot N_{0}\cdot N_{p}(t)$ | (S26) |

1. Remove the senescence component, which assumes the conversion to senescence after radiation is either negligible or has a negligible effect on the model’s ability to characterize the data. This results in the model defined by Eqs. (S27) – (S29):

| $\begin{matrix} \frac{dN_{p}(t)}{dt}=(k_{p}-k_{ld}(t,D,N_{0}))\cdot\left(\frac{N_{p}(t)+N_{s}(t)}{\theta}+A\right)\cdot N_{p}(t)\cdot\left(1-\frac{N_{p}(t)+N_{s}(t)}{\theta}\right) \\ \text{ }-k_{ed}(t,D,N_{0})\cdot N_{p}(t) \end{matrix}$ | (S27) |
| --- | --- |
| $k_{ed}(t,D,N_{0})=\sum_{i=1}^{\begin{matrix} fraction \\ number \end{matrix}} (\alpha_{acute,N}\cdot N_{0}+1)\cdot k_{acute,D}\cdot D\cdot f_{DSB}(t^{i},D)$ | (S28) |
| $k_{ld}(t,D,N_{0})=\sum_{1}^{\begin{matrix} fraction \\ number \end{matrix}} (\alpha_{accum,N}\cdot N_{0}+1)\cdot k_{accum,D}\cdot D\cdot t^{i}\cdot e^{-r\cdot t^{i}}$ | (S29) |

The following table summarizes the results of applying each of the above eight models on the training data set.

| Model # | Number of free parameters | AIC value (9L) | AIC value (C6) |
| --- | --- | --- | --- |
| 1 | 7 | -32.24 | -442.63 |
| 2 | 6 | -446.35 | -332.10 |
| 3 | 6 | **-466.09** | **-565.97** |
| 4 | 5 | -448.45 | -383.65 |
| 5 | 6 | 53.75 | -407.41 |
| 6 | 6 | -70.16 | -303.63 |
| 7 | 5 | 51.66 | -102.61 |
| 8 | 5 | -239.32 | -146.62 |

The Table indicates that model 3 (i.e., the model which does not include *k_acute_*_,_*_D_*) is most frequently selected as the most parsimonious model for both the 9L and C6 cell line data by achieving the lowest AIC value. Thus, model 3 is selected as the model to be employed in the prediction component of the study.

**S3. Heterogeneous response observed in our data.**


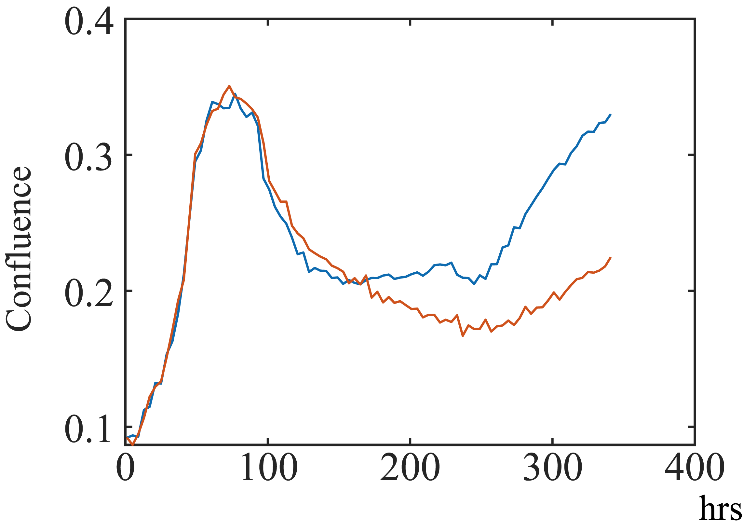


These are two replicates of C6 cell line with the same initial seeding density (~ 9% initial confluence) and under the same treatment (three fractions of 6.7 Gy). As our model doesn’t incorporate heterogeneous radiation sensitivity, it’s challenging to predict both curves accurately under the current framework. This will be the focus of a future study.
